# Dynamic Contrast-enhanced MRI in Renal Tumors: Common Subtype Differentiation using Pharmacokinetics

**DOI:** 10.1038/s41598-017-03376-7

**Published:** 2017-06-08

**Authors:** Hai-yi Wang, Zi-hua Su, Xiao Xu, Ning Huang, Zhi-peng Sun, Ying-wei Wang, Lu Li, Ai-tao Guo, Xin Chen, Xin Ma, Lin Ma, Hui-yi Ye

**Affiliations:** 1 Department of Radiology, Chinese PLA General Hospital, No.28 Fuxing Road, Haidian District, Beijing, 100853 China; 2 Beijing Aerospace Changfeng Co. Ltd., No. 51-A Yongding Road, Haidian District, Beijing, 100854 China; 3 Lift Science, Advanced Application Team, GE Healthcare China, Shanghai, 201203 China; 4 Lift Science, Advanced Application Team, GE Healthcare China, Beijing, 100176 China; 5 Department of Radiology, No.1 Hospital of Zhangjiakou, Zhangjiakou, 075000 Hebei Province China; 6 Department of Pathology, PLA General Hospital, Beijing, China, No. 28 Fuxing Road, Haidian District, Beijing, 100853 China; 7 Department of Urology, PLA General Hospital, Beijing, China, No. 28 Fuxing Road, Haidian District, Beijing, 100853 China

## Abstract

Preoperative renal tumor subtype differentiation is important for radiology and urology in clinical practice. Pharmacokinetic data (*K* ^trans^ & *V*_e_, etc.) derived from dynamic contrast-enhanced MRI (DCE-MRI) have been used to investigate tumor vessel permeability. In this prospective study on DCE-MRI pharmacokinetic studies, we enrolled patients with five common renal tumor subtypes: clear cell renal cell carcinoma (ccRCC; n = 65), papillary renal cell carcinoma (pRCC; n = 12), chromophobic renal cell carcinoma (cRCC; n = 9), uroepithelial carcinoma (UEC; n = 14), and fat-poor angiomyolipoma (fpAML; n = 10). The results show that *K* ^trans^ of ccRCC, pRCC, cRCC, UEC and fpAML (0.459 ± 0.190 min^−1^, 0.206 ± 0.127 min^−1^, 0.311 ± 0.111 min^−1^, 0.235 ± 0.116 min^−1^, 0.511 ± 0.159 min^−1^, respectively) were different, but *V*_e_ was not. *K* ^trans^ could distinguish ccRCC from non-ccRCC (pRCC & cRCC) with a sensitivity of 76.9% and a specificity of 71.4%, respectively, as well as to differentiate fpAML from non-ccRCC with a sensitivity of 100% and a specificity of 76.2%, respectively. Our findings suggest that DCE-MRI pharmacokinetics are promising for differential diagnosis of renal tumors, especially for RCC subtype characterization and differentiation between fpAML and non-ccRCC, which may facilitate the treatment of renal tumors.

## Introduction

The differentiation of renal tumor subtypes is important for radiologists and urologists in clinical practice, as each subtype is associated with a different tumor behavior and different prognosis. Although magnetic resonance imaging (MRI) is useful for differentiating between benign and malignant tissues^1–3^ and characterizing renal cell carcinoma (RCC) subtypes^4–6^, it cannot be used to accurately distinguish renal oncocytoma﻿s from RCCs^7, 8^, or renal angiomyolipomas with little fat from RCCs^9, 10^ or central RCCs from renal pelvic urothelial carcinomas^11^. Because benign lesions occur in ~20% of renal tumors^12, 13^, better preoperative diagnostic accuracy is needed.

Dynamic contrast-enhanced MRI (DCE-MRI) is routinely used for abdominal MRI and can provide enhanced information about dynamic changes in targeted lesions. When combined with unenhanced images, DCE-MRI can be used to characterize cystic degeneration or necrosis within lesions, facilitating accurate tumor diagnosis and the documentation of therapeutic outcomes. For renal tumor characterization, DCE-MRI can discriminate between benign and malignant renal lesions^1, 14^ and RCC subtypes^15, 16^. However, conventional DCE-MRI is subjective and depends upon lesion signal intensity that is visually defined.

Pharmacokinetic models^17^ that depict the distribution and diffusion of contrast agents between the vascular plasma space and the extravascular extracellular space (EES) can be used with DCE-MRI to quantify tumor growth and vascular permeability^18^. In addition, a DCE-MRI pharmacokinetic model can reflect tumor perfusion, vascular volume, and angiogenesis. It also allows the quantification of the volume transfer constant from plasma to the EES (*K* ^trans^), the efflux rate constant from EES back to plasma (*K*_ep_), the ratio of the EES volume to tissue volume (*V*_e_), and the ratio of blood plasma volume to tissue volume (*V*_p_).

In previous studies, DCE-MRI pharmacokinetics were chiefly used for the central nervous system and fixed organs. Specifically, *K* ^trans^ was used to evaluate histologic grades of intracranial gliomas^19^, and time-signal intensity curves of breast tumors combined with *K* ^trans^ were used to improve the diagnostic accuracy of breast carcinoma^20^. For renal tumors, DCE-MRI pharmacokinetics have been focused on the qualitative diagnosis and evaluation of targeted molecular therapy of metastatic or advanced RCC. However, no comprehensive quantitative analysis has been published. In a previous study, we confirmed that DCE-MRI pharmacokinetic data (*K* ^trans^ & *V*_e_) were reproducible in RCC^21^. Thus, in this study, we used DCE-MRI to perform pharmacokinetic assessments of common renal tumors, and investigated the value of using these pharmacokinetic data for the differentiation of renal tumor subtypes.

## Methods

### Patients

The Institutional Review Board of Chinese PLA General Hospital (Beijing, China) approved this study (#S2012-049-01). Study methods were performed in accordance with the Declaration of Helsinki, and written informed consent was obtained from each subject prior to study initiation. Patients with a renal tumor diagnosis were consecutively enrolled from September 2012 to December 2013 and underwent DCE-MRI scans using a 3.0 Tesla MR system (GE Discovery MR 750, GE Healthcare, Milwaukee, WI, USA). Inclusion criteria were as follows: >18 years of age; glomerular filtration rate (GFR) ≥60 mL/min, maximal renal tumor diameter ≥1 cm; pathologic tumor types including clear cell RCC (ccRCC), papillary RCC (pRCC), chromophobic RCC (cRCC), renal pelvic carcinoma (RPC), oncocytoma, and fat-poor renal angiomyolipoma (fpAML) (no fat attenuation on CT or fat signal intensity on MRI). Exclusion criteria included lesions with complete cystic degeneration or necrosis, poor imaging quality (cannot meet imaging analysis requirements); common contraindications for enhanced MRI such as allergy to gadolinium-related contrast agent, metal implants, or claustrophobia.

### MRI acquisition

All of the patients underwent an MRI scan within 48 h of the initial diagnosis. MRI examinations were performed on a 3.0 T scanner with a maximum gradient strength of 50 mT and maximum slew rate of 200 mT/s, using an 8-channel surface phased-array coil. Patients practiced breathing techniques before each scan, which included breathing quickly during a non-scanning break and then breath-holding in the same position for as long as possible. Routine clinical axial and coronal T2-weighted imaging was performed for all patients prior to dynamic studies to localize and delineate tumors. The imaging protocol for DCE-MRI consisted of a pre-contrast T1 mapping sequence and a DCE sequence. The former included five consecutive axial 3D spoiled-gradient recalled-echo sequences for liver acquisition with volume acceleration (LAVA) with an array of flip angles (3°, 6°, 9°, 12°, and 15°) in breath-hold mode. It also included an axial DCE sequence (flip angle, 12°): scanning during 12 s of breath-holding for two phases and a subsequent 6 s of breathing was performed repeatedly for up to 4.4 min to monitor contrast passage. Scanning parameters were as follows: repetition time (TR), 2.8 ms; echo time (TE), 1.3 ms; matrix, 288 × 180; field of view (FOV), 38 × 38 cm; slice thickness, 6 mm; number of excitations (NEX), 1; bandwidth, 125 kHz; and parallel imaging acceleration factor, 3. The contrast agent, gadodiamide (0.1 mmol/kg, Omniscan, GE Healthcare) was given intravenously when the second scan was started at a rate of 2 mL/s using a power injector (Spectris; MedRad, Warrendale, PA, USA). The contrast bolus was flushed with 20 mL normal saline, administered at the same rate, to improve bolus coherence.

### Image post-processing and analysis

All of the DCE-MRI analyses were conducted using open-source software packages, including the R package (http://dcemri.sourceforge.net/) and a medical image non-rigid registration package (http://cmictig.cs.ucl.ac.uk/wiki/index.php/NiftyReg).

All images were transferred to an Omni-Kinetics workstation (GE Healthcare, LifeScience, China) for analysis. The breath-hold position for each patient differed and the shape of the kidney non-rigidly varied between individuals. It has been shown that image registration methods^22^ can be used to handle body motion within the time domain^23, 24^. Here, the workstation provided an automatic nonlinear registration framework^25^ to remove errors of misalignment between consecutive MRI scans, thereby increasing accuracy. The registration framework used a free-form deformation algorithm^26^ as the main registration engine and mutual information as the correspondence metric^27^.

### Data Collection

#### Calculation of pharmacokinetic parameters

A multiple flip angle method^17, 28^ was used to perform T1 mapping to obtain the T1 value of the tissue before and after contrast agent injection. Then the contrast agent concentration in the tissue was computed using tissue signal intensity. A two-compartment extended-Tofts model was used^29^ (Eq. 1) with a population-based arterial input function (AIF)^17, 28^ (Eq. 2) to calculate parameters. In Equation 1, *K* ^trans^ is the transfer constant from plasma to the EES, *V*_e_ is the ratio of EES volume to tissue volume, *V*_p_ is the ratio of blood plasma volume to tissue volume, *K*_ep_ = *K* ^trans^/*V*_e_ is the efflux rate constant from EES to plasma, and *C*_*t*_(*t*) and *Cp*(*t*) represents the contrast agent concentrations in the tissue and plasma, respectively. In Equation 2, D = 1.0 mmol/kg, a_1_ = 2.4 kg/l, a_2_ = 0.62 kg/l, m_1_ = 3.0, and m_2_ = 0.016.

1 $${C}_{t}(t)={K}^{trans}{\int }_{0}^{t}{C}_{p}(\tau ){e}^{-\frac{{K}^{trans}}{{V}_{e}}(t-\tau )}d\tau +{V}_{p}\cdot {C}_{p}(t)$$

2 $${C}_{p}(t)=D({{\rm{a}}}_{1}{{\rm{e}}}^{-{m}_{1}t}+{{\rm{a}}}_{2}{{\rm{e}}}^{-{m}_{2}t})$$

### Region of interest selection

All of the images were transferred to a Sun workstation (Sparc 10, Sun Microsystems, Mountain View, CA, USA), at which pharmacokinetics were measured using ImageJ software (National Institutes of Health, Bethesda, MD, USA). Using reference information from anatomic axial and coronal T2-weighted images and post-contrast T1 images, one radiologist blinded to the pathologic results was instructed to place region of interests (ROIs) on the slice with the largest diameter of tumors according to dynamic images of DCE-MRI, covering the whole tumor where possible but excluding pulsatile artifacts from blood vessels and susceptibility artifacts from adjacent bowels. Then, the same ROI was copied to parametric maps (*K* ^trans^, *V*_e_).

### Pathologic analysis

All of the specimens after partial nephrectomy or radical nephrectomy were examined by two uropathologists blinded to MRI findings, and the consensus was used for final decisions. All of the lesions were pathologically characterized according to World Health Organization tumor classification of the kidney^30^. Hematoxylin and eosin (H&E) staining and immunohistochemistry for cluster of differentiation 31 (CD31) were performed.

### Statistical Analyses

#### Test of Normality

Normality of all data was analyzed using Shapiro-Wilk method. Normality was confirmed when *p* > 0.05.

#### Differences in the pharmacokinetics of renal tumor subtypes

All of the pharmacokinetics are expressed as mean ± standard deviation (SD) or median with ranges. Differences in the pharmacokinetics of different renal tumor subtypes were evaluated using an independent samples Kruskal-Wallis test or a one-way analysis of variance (ANOVA).

#### Difference in pharmacokinetics between benign and malignant tumors

CcRCC, pRCC, cRCC, and UEC were classified as malignant tumors and fpAML and oncoctyoma were defined as benign. Pharmacokinetic differences between benign and malignant tumors were evaluated using an independent samples *t*-test or a Mann-Whitney U test.

#### Difference in pharmacokinetics of RCC subtypes

Pharmacokinetic differences among RCC subtypes were evaluated using an independent samples Kruskal-Wallis test or one-way ANOVA and differences between ccRCC and non-ccRCC (pRCC and cRCC) were analyzed using an independent samples *t*-test or Mann-Whitney U test. A receiver operating characteristic (ROC) curve was used to analyze the diagnostic sensitivity and specificity, and to calculate Youden’s index.

#### Differences in pharmacokinetics between fpAML and non-ccRCCs and between RCCs and UECs

Pharmacokinetic differences between fpAML and non-ccRCCs and between RCCs and UECs were evaluated using an independent samples *t*-test or Mann-Whitney U test, and an ROC was used to analyze the diagnostic sensitivity and specificity, and to calculate Youden’s index. All of the statistical analyses were performed with SPSS software (IBM SPSS Statistics for Macintosh, Version 22.0. IBM Corp., Armonk, NY, USA) and *p* values less than 0.05 were considered statistically significant. However, for multiple samples compared with ANOVA or the Kruskal-Wallis test, *p* values less than 0.01 were considered statistically significant.

## Results

### Patient information and lesion characterization

**Figure 1:**
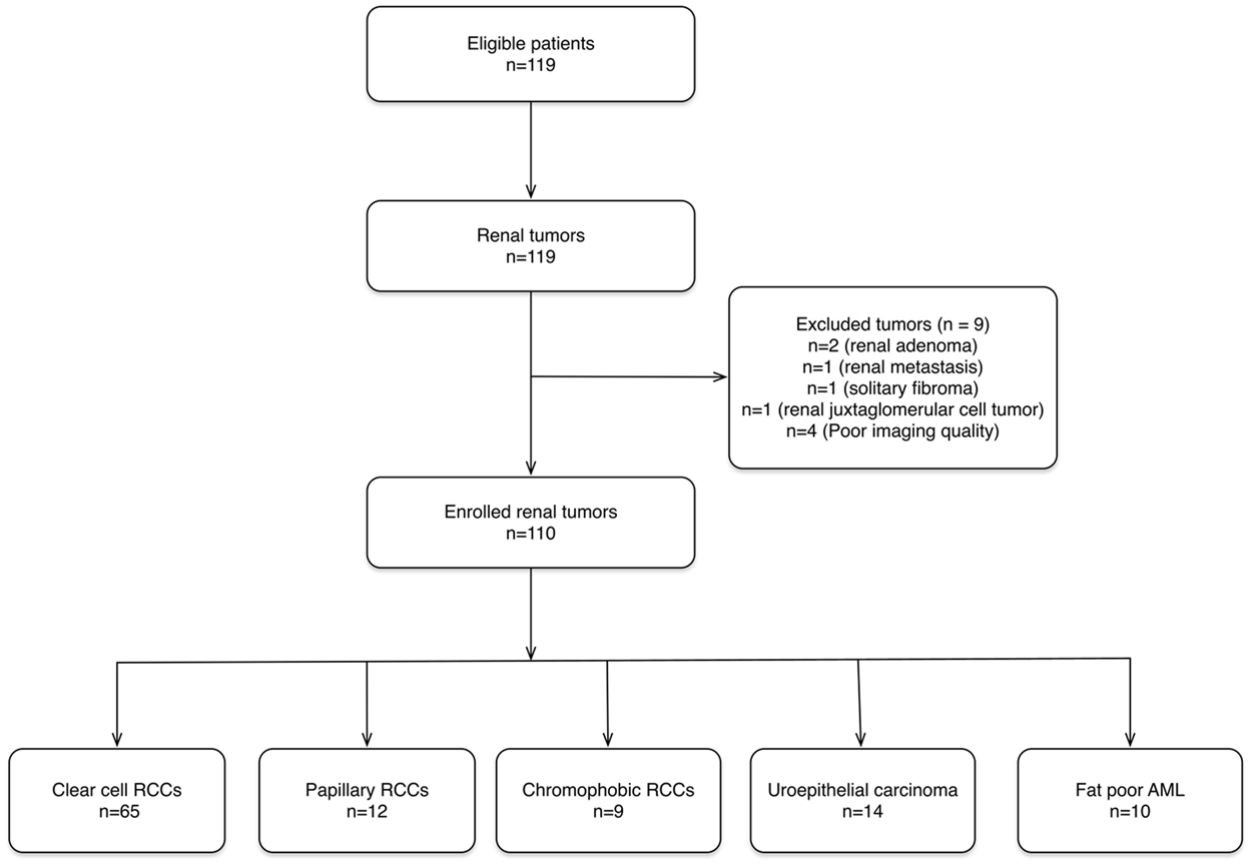
Flowchart of study enrollment. RCC = renal cell carcinoma, AML = angiomyolipoma.

Patient information, surgical and pathologic data were collected by a senior attending radiologist, and data for the subjects appear in Table 1. Of the enrolled subjects, 82 patients underwent partial nephrectomy and 37 underwent radical nephrectomy. The interval between DCE-MRI scanning and surgery was 7.2 ± 3.8 days. After excluding renal adenoma (n = 2), renal metastasis (n = 1), solitary fibroma (n = 1), juxtaglomerular cell tumor (n = 1), and cases with poor imaging quality (n = 4), a total of 110 patients underwent DCE-MRI pharmacokinetic analysis (Fig. 1). We did not enroll patients with oncocytomas.

**Table 1:** Patient information and pathologic results (n = 110).

	ccRCC	pRCC	cRCC	UEC	fpAML
Sex (Male/Female)	48/17	9/3	3/6	6/8	3/7
Age (Years) (Mean age ± SD)	52.9 ± 10.4	52.3 ± 11.1	48.9 ± 15.3	62.3 ± 8.2	52.1 ± 11.7
Number of Tumor	65	12	9	14	10
Size of Tumor (cm) (Mean maximum diameter ± SD)	4.2 ± 1.8	3.2 ± 1.3	4.2 ± 2.0	3.1 ± 0.9	3.8 ± 1.2

Note: ccRCC, pRCC, cRCC, UEC, fpAML represent clear cell renal cell carcinoma, papillary renal cell carcinoma, chromophobic renal cell carcinoma, uroepithelial carcinoma, and fat poor angiomyolipoma, respectively.

### Test of Normality

According to the Shapiro-Wilk test, *K* ^trans^ values for ccRCC, cRCC, and UEC were normally distributed, but *K* ^trans^ values for pRCC and fpAML were not. *V*_e_ values for each group were normally distributed (*p* > 0.05).

### Comparison of DCE-MRI pharmacokinetics among renal tumor subtypes

**Figure 2:**
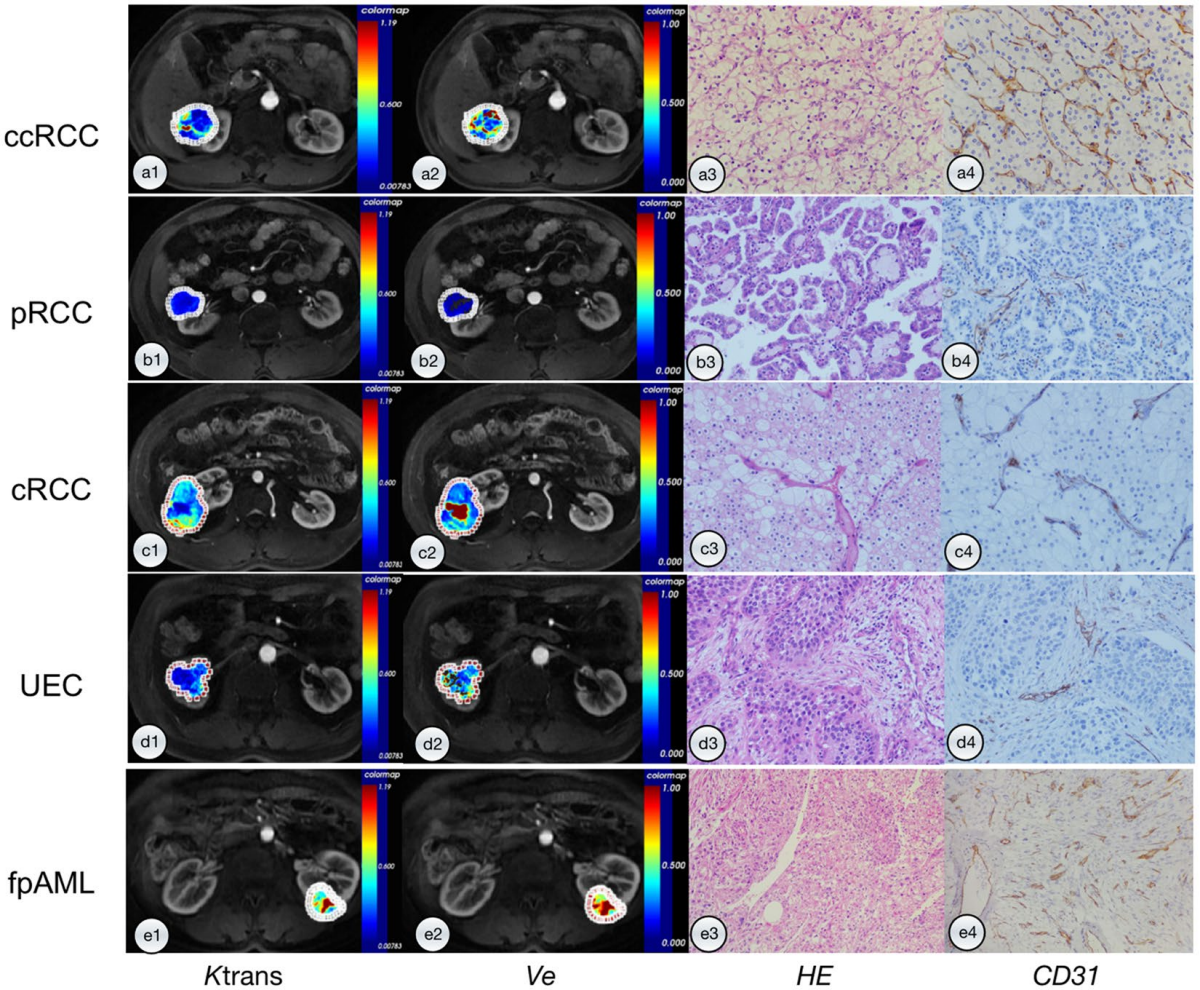
*K* ^trans^, *V*_e_ maps and photomicrographs of five renal tumor types. (**a**) A 46-year-old male with ccRCC. (a1) *K* ^trans^ value of ccRCC was 0.372 min^−1^; (a2) *V*_e_ value of ccRCC was 0.497; (a3) H&E staining (original magnification, 20×) shows solid alveolar and acinar patterns arranged by tumor cells with a clear cytoplasm and a regular network of small thin-walled blood vessels.; (a4) Immunohistochemistry for CD31 (original magnification, 20×) shows a network of small, thin-walled blood vessels. (**b**) A 45-year-old male with pRCC. (b1) *K* ^trans^ value of pRCC was 0.094 min^−1^; (b2) *V*_e_ value of pRCC was 0.213; (b3) H&E staining (original magnification, 20×) shows papillae formed by delicate fibrovascular cores that contain foamy macrophages. Papillae are lined with cytologically low-grade cells with a small amount of cytoplasm and relatively uniform nuclei; (b4) Immunohistochemistry CD31 (original magnification, 20×) shows small, delicate blood vessels in the fibrovascular cores, but not between the papillae. (**c**) A 49-year-old male with cRCC. (c1) *K* ^trans^ value of cRCC was 0.259 min^−1^; (c2) *V*_e_ value of cRCC was 0.439; (c3) H&E staining (original magnification, 20×) shows tumor cells arranged in a solid sheet-like pattern, separated by incomplete, hyalinized vascular septa. Tumor cells are round to polygonal and have well-defined cytoplasmic borders, pale eosinophilic cytoplasm with a fine reticular pattern, and perinuclear halos; (c4) Immunohistochemistry CD31 (original magnification, 20×) shows positively stained endothelial cells lining the surface of incomplete and hyalinized vascular septa. (**d**) A 67-year-old male with UEC. (d1) *K* ^trans^ value of UEC was 0.203 min^−1^; (d2) *V*_e_ value of UEC was 0.498; (d3) H&E staining (original magnification, 20×) shows infiltrating cohesive nests of cells with moderate-to-abundant amphophilic cytoplasm and large hyperchromatic nuclei. Irregular blood vessels of different sizes and thicknesses in stroma between tumor cell nests; (d4) Immunohistochemistry CD31 (original magnification, 20×) shows positively stained endothelial cells lining irregular blood vessels in the stroma. (**e**) A 46-year-old female with fpAML. (e1) *K* ^trans^ value of fpAML was 0.607 min^−1^; (e2) *V*_e_ value of fpAML was 0.570; (e3) H&E (original magnification, 20×) shows tumor composed of various mixtures of thick-walled and poorly organized blood vessels, smooth muscle and rare mature fat; (e4) IHC CD31 (original magnification, 20×) shows thick-walled poorly organized blood vessels.

**Figure 3:**
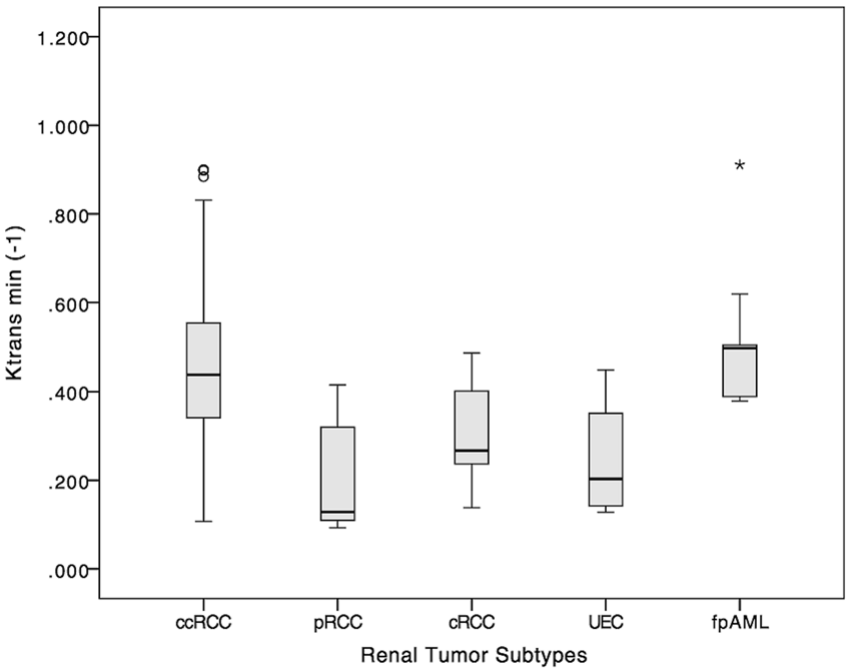
Box-and-whisker plot of *K* ^trans^ value. Boxes = interquartile range, whiskers = range of all values, horizontal line within box = median *K* ^trans^, ccRCC = clear cell RCC, pRCC = papillary RCC, cRCC = chromophobic RCC, UEC = uroepithelial carcinoma, fpAML = fat poor angiomyolipoma.

**Figure 4:**
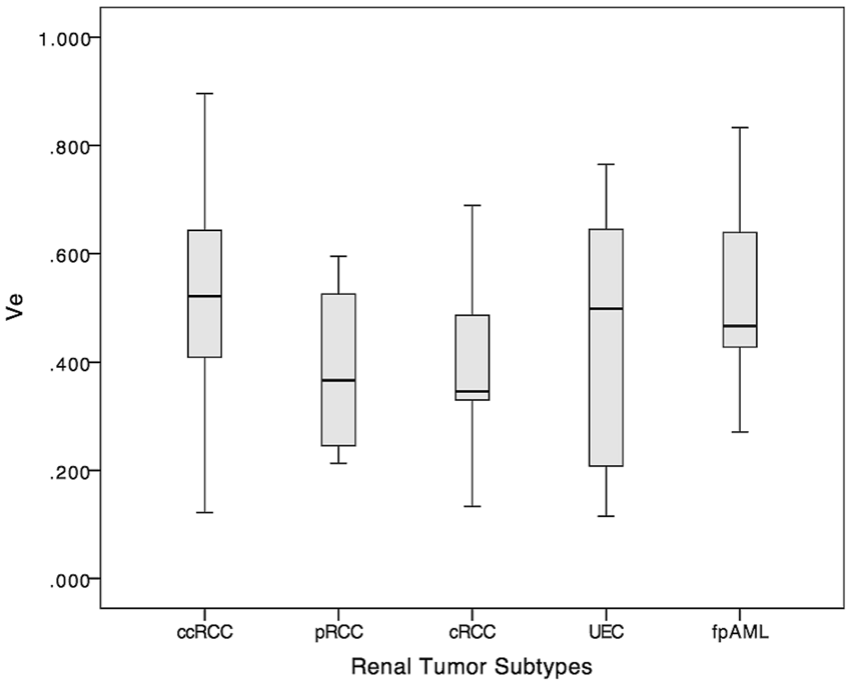
Box-and-whisker plot of *V*_e_ value. Boxes = interquartile range, whiskers = range of all values, horizontal line within box = median *V*_e_, ccRCC = clear cell RCC, pRCC = papillary RCC, cRCC = chromophobic RCC, UEC = uroepithelial carcinoma, fpAML = fat poor angiomyolipoma.

The *K* ^trans^ and *V*_e_ parametric maps of five renal tumor subtypes are shown in Fig. 2. Differences in *K* ^trans^ among five renal tumors were statistically significantly different (*p* < 0.001) and pairwise comparisons appear in Table 2 and Fig. 3. Differences in *V*_e_ among the five renal tumors were not statistically significantly different (*p* = 0.044; Fig. 4).

**Table 2:** Pairwise comparison of DCE-MRI pharmacokinetics of renal tumors.

Pairwise	*P* Value (*K* ^trans^)	*P* Value (*V*_e_)
1 vs 2	<0.001	0.014
1 vs 3	0.034	0.039
1 vs 4	<0.001	0.137
1 vs 5	0.256	0.770
2 vs 3	0.160	0.929
2 vs 4	0.625	0.389
2 vs 5	<0.001	0.114
3 vs 4	0.318	0.483
3 vs 5	0.013	0.165
4 vs 5	<0.001	0.411

Note: 1: clear cell renal cell carcinoma; 2: papillary renal cell carcinoma; 3: chromophobic renal cell carcinoma; 4: uroepithelial carcinoma; 5: fat-poor angiomyolipoma.

### Comparison of DCE-MRI pharmacokinetics between benign and malignant tumors

The *K* ^trans^ values for renal malignant tumors and benign tumors (fpAML) were 0.393 ± 0.193 min^−1^ and 0.511 ± 0.159 min^−1^, respectively. Differences in *K* ^trans^ of renal malignant tumors and benign tumors (fpAML) were not statistically significant (*p* = 0.064) nor was the *V*_e_ of renal malignant tumors and benign tumors (fpAML) (*p* = 0.721) (Table 2).

### Comparison of DCE-MRI pharmacokinetics among RCC subtypes

**Figure 5:**
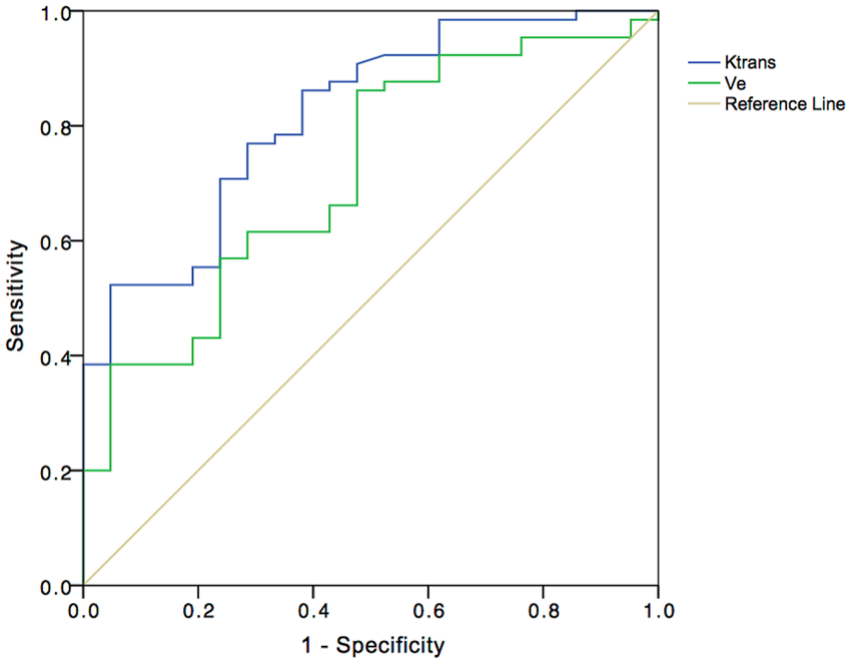
ROC curve of *K* ^trans^ (blue line) and *V*_e_ (green line) shows comparisons of *K* ^trans^ and *V*_e_ in ccRCC and non–ccRCCs. With a *K* ^trans^ value of 0.330 min^−1^, sensitivity and specificity were 76.9% and 71.4%, respectively, and the AUC was 0.819. With a *V*_e_ of 0.327, sensitivity and specificity were 86.2% and 52.4%, respectively, and the AUC was 0.716.

The *K* ^trans^ of ccRCC and non-ccRCC (0.459 ± 0.190 min^−1^ and 0.251 ± 0.130 min^−1^, respectively) was statistically significantly different (*p* < 0.001). Threshold *K* ^trans^ values that could distinguish ccRCC from non-ccRCC are shown in Fig. 5 along with specificity and sensitivity (Youden’s index 0.483), and the AUC data. *V*_e_ data for ccRCC and non-ccRCC were statistically significantly different (*p* = 0.002) and the cutoff *V*_e_ values to distinguish ccRCC from non-ccRCC are shown in Fig. 5 along with the sensitivity and specificity (Youden’s index 0.386) and AUC data.

### Comparison of DCE-MRI pharmacokinetics between fpAML and non-clear cell RCCs

**Figure 6:**
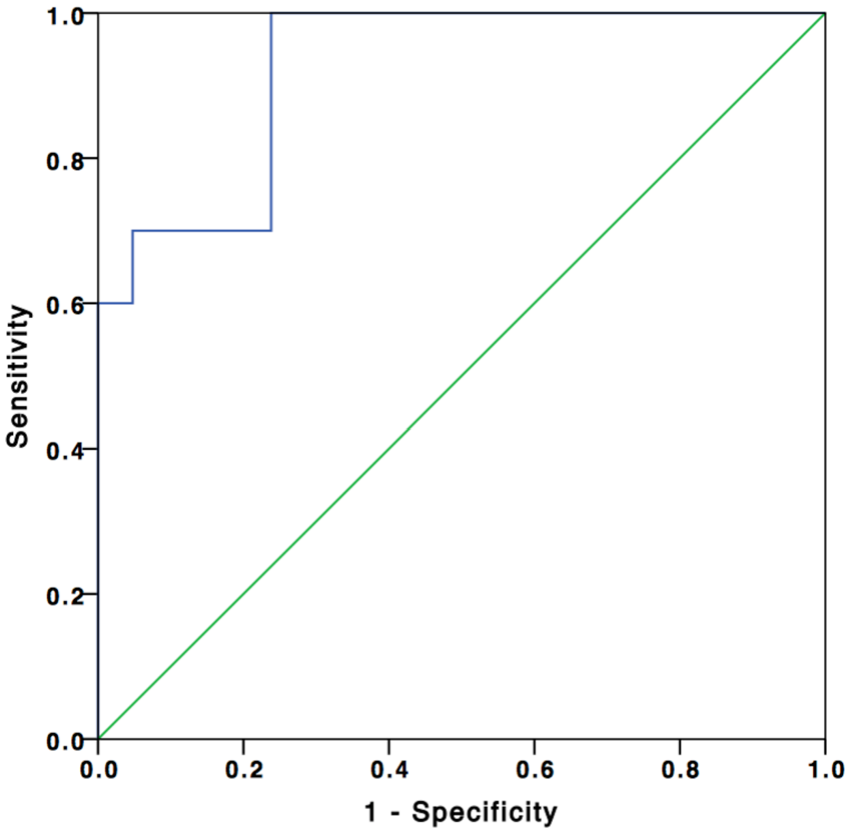
ROC curve (blue line) comparison of *K* ^trans^ in fpAML and non-ccRCCs. The AUC was 0.924. When the threshold *K* ^trans^ value was 0.365 min^−1^, the sensitivity and specificity were 100% and 76.2%, respectively. When the *K* ^trans^ value was greater than 0.427 min^−1^, the sensitivity and specificity were 70.0% and 95.2%, respectively.

*K* ^trans^ values for fpAML and non-ccRCCs were statistically significantly different (*p* < 0.001). Threshold *K* ^trans^ values to distinguish fpAML from non-ccRCCs as well as sensitivity and specificity (Youden’s index 0.762) and AUC data appear in Fig. 6. *V*_e_ values for fpAML and non-ccRCCs were not statistically significantly different (*p* = 0.069).

### Comparisons of DCE-MRI pharmacokinetics between RCC and UEC

**Figure 7:**
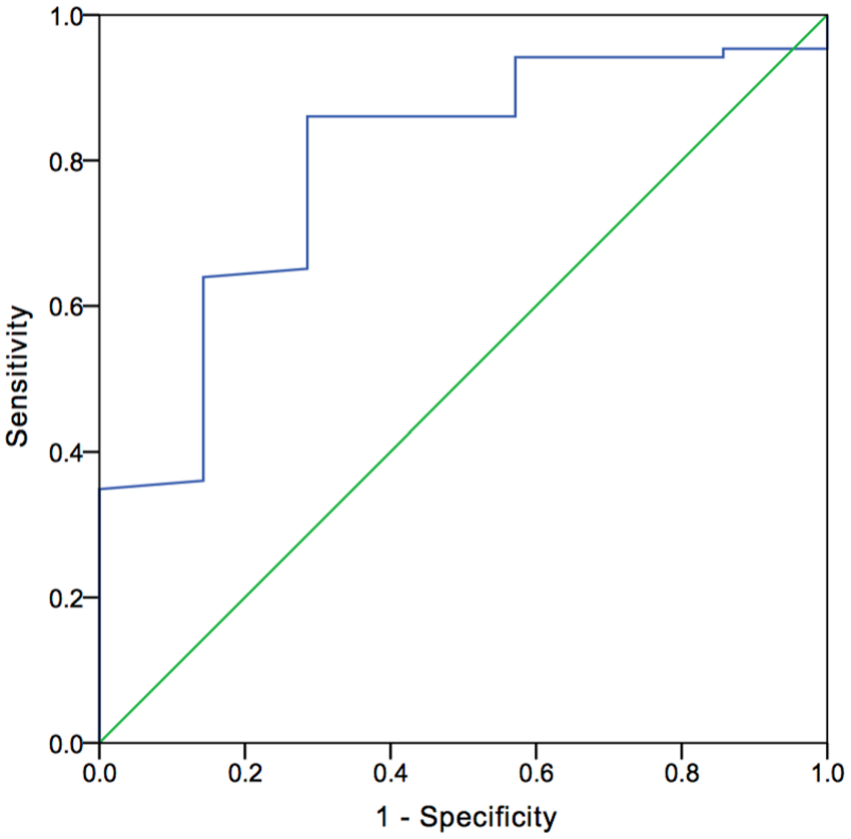
ROC curve (blue line) comparing *K* ^trans^ in RCCs and UECs. The AUC was 0.766. When the threshold *K* ^trans^ value was 0.563 min^−1^, the sensitivity and specificity were 84.9% and 71.4%, respectively.

*K* ^trans^ of RCCs and UECs were statistically significantly different (*p* = 0.015). Threshold *K* ^trans^ values to distinguish RCC from UEC appear in Fig. 7 along with sensitivity and specificity data (Youden’s index 0.762). AUC data appear in Fig. 7 as well. *V*_e_ for RCCs and UECs were not statistically significantly different (*p* = 0.396).

## Discussion

The accurate diagnosis of renal masses can be accomplished by analyzing the imaging features of renal masses. Although diagnostic imaging is often used to diagnose renal masses, it comes with a number of challenges. Thus, in this study, we used DCE-MRI pharmacokinetics to characterize renal masses among five renal tumor subtypes to determine if kinetic measurements could be used as an alternative diagnostic tool for the differential diagnosis of renal tumors. Of these, fpAML had the greatest *K* ^trans^ followed by ccRCC, cRCC, UEC, and pRCC. The fpAML and ccRCC values were not different statistically but the *K* ^trans^ of ccRCC was greater than that of pRCC, a finding that is in accordance with the literature^31^. fpAML had the greatest *K* ^trans^, likely due to its thick-walled blood vessels that lack arterial elasticity^30, 32^. ccRCC tumors have a rich and regular network of small thin-walled blood vessels, which may create high *K* ^trans^. pRCC tumors have few blood vessels, which may contribute to the low *K* ^trans^.

Using *K* ^trans^ and *V*_e_ to distinguish between renal benign and malignant tumors produced no statistically significant differences, which may be explained by the fact that ccRCCs accounted for most of the malignant tumors and their pharmacokinetics were similar to those of fpAMLs. For ccRCC and non-ccRCC, *K* ^trans^ was statistically significantly different and *K* ^trans^ had a large area under the ROC curve for diagnosing ccRCC (0.819); however, the *V*_e_ values were not significantly different.

Differentiating fpAML from non-ccRCC is of interest, but previous studies have shown that CT is of little value in this regard^33^ and that fpAML and pRCC often overlap in images^34, 35^; specifically, both renal masses can appear hypointense on T2- weighted images. Although MRI has been used to analyze imaging differences between fpAML and RCCs^10, 36, 37^, the analyses were done by grouping ccRCC and non-ccRCC together instead of analyzing them separately, the latter of which is the ideal way to analyze these two different types of tumors^10, 38^. ccRCCs have many distinguishing features compared to fpAML, so positive results would be expected. Here, we focused on the differentiation between fpAML and non-ccRCCs, and noted that *K* ^trans^ was statistically significantly different between these tumor subtypes, with an area under the ROC curve of 0.924. When the threshold for *K* ^trans^ of 0.365 min^−1^ was selected, the sensitivity and specificity of fpAML were high. Increasing the threshold *K* ^trans^ value to 0.427 min^−1^, improved specificity and worsened sensitivity, which may allow preoperative distinctions between fpAML and non-ccRCC.

Uroepithelial carcinoma of the renal pelvis or renal pelvic carcinoma that invades the renal parenchyma may mimic RCC in the center of the kidney. Wehrli’s group^11^ pointed out that T2 weighted image signal intensity and uncorrected apparent diffusion coefficient values were not different between RPC and RCC. We observed that RCCs had larger *K* ^trans^ than RPCs, likely because RCCs have a higher microvascular density than RPCs. With a threshold of 0.228 min^−1^, *K* ^trans^ can distinguish RCCs from RPC (AUC 0.766; sensitivity 86%; specificity 71.4%), which may be useful for distinguishing between these tumors. However, *V*_e_ was not different between RCC and RPC, so this value cannot be used as a distinguishing index.

For the DCE-MRI technique, we chose a population averaged arterial input function (AIF) instead of a personal AIF to perform pharmacokinetic calculations. Personal or individual AIFs, if calculated accurately, can improve pharmacokinetic studies, but personal AIFs require a high temporal resolution and may be influenced by physiology, ROI placement, partial volume effects, and inflow effects. Due to the non-continuous scanning mode of the DCE-MRI (See “MRI technique” in Methods) for balancing clinical practice and scientific research needs, the temporal resolution of DCE-MRI was limited. Thus, we used a population-based AIF method, which addressed temporal resolution difficulties and reduced AIF ROI location and sizing errors as previously reported^39^. In addition, population-based AIF works as well as individual AIF for estimating pharmacokinetics, as confirmed by several investigators^40–42^.

The limitations of this study include the necessity of image registration and establishment of kinetic parametric maps, which was time-consuming and is not ideal for clinical practice. Thus, more user-friendly software or an accelerating method should be investigated. Second, ROIs covering the entire tumor on the slice with its maximum diameter was the most reproducible method for drawing ROI in DCE-MRI analysis, but this method ignores necrosis, cystic changes, and hemorrhages, which may induce errors in analysis. In the future, histogram analysis of pharmacokinetics should be attempted. Third, we were unable to enroll patients with renal oncocytoma, as this is a relatively rare disease, making it difficult to obtain an appreciable sample size. Previous work indicates that oncocytoma has a similar *K* ^trans^ and *V*_e_ as ccRCC^31^, but our sample size was small (n = 3). Thus, additional research is required to validate our findings. Finally, a few patients in our center chose CT instead of MRI for imaging, and of those who agreed to undergo MRI examination, many could not endure the lengthy DCE-MRI process.

In conclusion, DCE-MRI kinetic measurements are promising for the differential diagnosis of renal tumors, especially for RCC subtype characterization, and for distinguishing between fpAML and non-ccRCC.
